# Supplementary material for: Hybrid Models and Biological Model Reduction with PyDSTool
Source: PLoS Comput Biol. 2012 Aug 9;8(8):e1002628. doi: 10.1371/journal.pcbi.1002628 (PMC3415397; doi:10.1371/journal.pcbi.1002628)
Supplement: Text S4 — Complete source code for the PyDSTool package (version 0.88.120504). Includes API documentation and help files linking to web pages. This file is identical to the current public release on Sourceforge.net. (ZIP) [file pcbi.1002628.s004.zip › PyDSTool/html/PyDSTool.Model.boundary_containment_by_postproc-class.html]

xml version="1.0" encoding="ascii"?


PyDSTool.Model.boundary\_containment\_by\_postproc


| Home | Trees | Indices | Help | PyDSTool |
| --- | --- | --- | --- | --- |

Package PyDSTool ::
Module Model ::
Class boundary\_containment\_by\_postproc

[hide private]

[frames] | no frames]

# Class boundary\_containment\_by\_postproc

source code

```
       object --+                
                |                
 MProject.feature --+            
                    |            
MProject.feature_leaf --+        
                        |        
 MProject.qt_feature_leaf --+    
                            |    
         boundary_containment --+
                                |
                               boundary_containment_by_postproc
```

---


|  |  |
| --- | --- |
| Instance Methods | [hide private] |

|  |  |
| --- | --- |
| evaluate(self, traj) | source code |

|  |  |
| --- | --- |
| \_find\_idx(self)  Helper function for finding index in trajectory meshpoints at which containment first failed | source code |

**Inherited from `boundary_containment`**:
`__init__`

**Inherited from `MProject.feature_leaf`**:
`__repr__`,
`__str__`,
`set_ref_traj`

**Inherited from `MProject.feature_leaf`** (private):
`_residual_info`

**Inherited from `MProject.feature`**:
`__call__`,
`__eq__`,
`__hash__`,
`__ne__`,
`finish`,
`info`,
`postprocess_ref_traj`,
`prepare`,
`reset_metric`,
`validate`

**Inherited from `object`**:
`__delattr__`,
`__getattribute__`,
`__new__`,
`__reduce__`,
`__reduce_ex__`,
`__setattr__`


|  |  |
| --- | --- |
| Properties | [hide private] |

**Inherited from `object`**:
`__class__`


|  |  |
| --- | --- |
| Method Details | [hide private] |

|  |  |
| --- | --- |
| evaluate(self, traj) | source code |

Overrides: MProject.feature.evaluate

|  |  |
| --- | --- |
| \_find\_idx(self) | source code |

Helper function for finding index in trajectory meshpoints at which
containment first failed

Overrides: MProject.feature.\_find\_idx

  


| Home | Trees | Indices | Help | PyDSTool |
| --- | --- | --- | --- | --- |

|  |  |
| --- | --- |
| Generated by Epydoc 3.0.1 on Fri May 4 15:24:07 2012 | http://epydoc.sourceforge.net |
